# Waning immune response to CoronaVac in health care workers from different age groups

**DOI:** 10.47626/1679-4435-2022-1048

**Published:** 2024-09-24

**Authors:** Monica Taminato, Ana Paula Chaves, Richarlisson Morais, Danielle Conte, Maria Cristina Gabrielloni, Gabriela Barbosa, Eduardo Medeiros, Nancy Bellei

**Affiliations:** 1 Medicina, Universidade Federal de São Paulo, São Paulo, SP, Brazil

**Keywords:** COVID-19 vaccines, COVID-19 serological testing, SARS-CoV-2, antibodies, healthcare workers, vacinas contra COVID-19, teste sorológico para COVID-19, SARS-CoV-2, anticorpos, trabalhadores da saúde

## Abstract

**Introduction:**

The waning of serum antibodies against severe acute respiratory syndrome coronavirus 2 observed in several studies raises questions about long-term immunity. Lower antibody levels are associated with new cases of COVID-19 even postvaccination, leading to the administration of booster doses.

**Objectives:**

To evaluate the postvaccination immune humoral response and the relationship between postvaccination seropositivity rates and demographic data among health care workers 6 months after CoronaVac vaccination.

**Methods:**

This was a cross-sectional study including health care workers vaccinated with two doses of CoronaVac after 6 months or more. The present study was conducted with the analysis of postvaccination serology test to assess the level of humoral response (anti-receptor binding domain IgG) after vaccination.

**Results:**

A total of 325 participants were enrolled, of whom 76% were female, with a median age of 42 years (20-85; interquartile range 31-53). Overall, 18.8% (61) of the participants results were seropositive for anti-receptor binding domain IgG; 81.2% did not have sufficient quantitative titers. The IgG titers obtained from female health care workers did not differ from those obtained from seropositive male health care workers, regardless of age.

**Conclusions:**

A group of positive quantitative titers was identified in the serology test for IgG antibodies against severe acute respiratory syndrome coronavirus 2. Further studies are needed to determine the durability of postvaccination antibodies and how serology testing can be used to determine the ideal timing for booster doses of the vaccine.

## Abstract (pt)

**Introdução:**

O declínio dos anticorpos séricos contra a síndrome respiratória aguda grave do coronavirus 2 observado em vários estudos levanta questões sobre a imunidade a longo prazo. Níveis mais baixos de anticorpos estão associados a novos casos de covid-19 mesmo após a vacinação, levando à administração de doses de reforço.

**Objetivos:**

Avaliar a resposta imunitária humoral após a vacinação e a relação entre as taxas de soropositividade após a vacinação e dados demográficos em trabalhadores da saúde por mais de 6 meses após a imunização com CoronaVac.

**Métodos:**

Estudo transversal incluindo profissionais de saúde vacinados com duas doses de CoronaVac após 6 meses ou mais. O estudo foi realizado com a análise do teste sorológico após a vacinação para avaliar os níveis de resposta humoral (anti-domínio de ligação ao receptor IgG) após a vacinação.

**Resultados:**

Neste estudo foram incluídos 325 participantes, 76% do sexo feminino e a idade mediana foi de 42 anos (20-85; intervalo interquartil 31-53). No geral, 18,8% (61) dos resultados dos participantes foram soropositivos para IgG anti-domínio de ligação ao receptor; 81,2% não apresentaram títulos quantitativos suficientes. Os títulos de IgG obtidos para os profissionais de saúde do sexo feminino não foram diferentes daqueles obtidos para os participantes do sexo masculino com soropositividade, independentemente da idade.

**Conclusões:**

Foi possível identificar um grupo com títulos quantitativos positivos no teste sorológico para anticorpo IgG contra a síndrome respiratória aguda grave do coronavírus 2. Mais investigações são necessárias para determinar a durabilidade dos anticorpos após a vacinação e como os testes sorológicos podem determinar o momento ideal das doses de reforço da vacina.

## INTRODUCTION

Severe acute respiratory syndrome coronavirus 2 (SARS-CoV-2) has infected millions of people worldwide. Brazil is one of the countries with the highest number of confirmed cases and deaths from SARS-CoV-2.^1,2,3^

CoronaVac (Sinovac Life Sciences, Beijing, China), an inactivated vaccine, was approved for emergency use by Agência Nacional de Vigilância Sanitária (ANVISA) in January 2021. It was first distributed shortly after approval on January 18, 2021.^4^ As one of the most affected groups in the COVID-19 pandemic, health care workers (HCW) were a priority group to be vaccinated. Two doses of CoronaVac vaccine were administered, with the recommended 28-day interval between the first and second doses. This interval was the most effective against the more serious outcomes of hospitalization, intensive care unit (ICU) admission, and death.^5^

Waning of serum antibodies against SARS-CoV-2 observed in several studies raises questions about long-term immunity. Lower antibody levels are associated with new cases of COVID-19 even postvaccination, leading to the administration of booster doses.^6,7,8^ Seropositivity for antibodies against SARS-CoV-2 might correlate with the risk of future infection over time, as studies have shown that neutralizing and binding antibodies have a strong correlation with efficacy.^6^

The Brazilian vaccination schedule was introduced in October 2021, providing a third dose for HCWs and persons aged 60 years and older. However, the persistence of CoronaVac-induced immunity is unknown, and immunogenicity may vary across age cohorts. The aim of the study was to evaluate the postvaccination immune humoral response as well as the relationship between postvaccination seropositivity rates and demographic data (i.e., age and sex) among HCWs after 6 months of two doses of CoronaVac.

## METHODS

We conducted a cross-sectional study of HCWs from Hospital São Paulo (state of São Paulo, Brazil) who were vaccinated with CoronaVac with at least 6 months after the administration of the second dose. The study was performed in October 2021 with an analysis of postvaccination IgG antibodies.

### SUBJECTS

HCWs at the Hospital São Paulo were invited to have their blood drawn for serology testing for quantitative anti-receptor binding domain (anti-RBD) IgG to assess the level of humoral response after at least 6 months of whole-virion CoronaVac vaccination. All HCWs who had received the second dose of CoronaVac vaccine within 6 months or more were eligible. Exclusion criteria were a previous infection with coronavirus disease 2019, immunosuppression, or use of immunosuppressive drugs. The study was approved by the research ethics committee of the Universidade Federal de São Paulo (certificate of ethical appraisal no. 47617621.6.0000.5505). All participants signed an informed consent form to participate in the study.

### LABORATORY STUDY

The study was performed in October 2021 at Laboratório de Virologia. A total of 3-5 mL of venous blood was taken from the volunteers participating in the study. Sera were separated and stored in a -20 °C freezer until antibody testing were performed. IgG antibody assays against SARS-CoV-2 RBD protein were performed by using the Access SARS-CoV-2 IgG antibody assay (1ºIS) (Beckman Coulter, Inc.). Antibodies against RBD of the spike protein were quantitatively analyzed and interpreted as positive (signal for test sample/signal at cutoff value if ≥ 30 UI/mL or binding antibodies units per mL [BAU/mL]) or negative (if ≥ 30 UI/mL) in accordance with the Access SARS-CoV-2 IgG antibody assay.

### STATISTICAL ANALYSIS

Data are presented as counts, percentages, and 95%CIs. Comparisons were performed between positive and negative groups according to the detection of IgG anti-RBD. The Shapiro-Wilk normality test was performed to verify normality, with a significance level of 5%. The Kruskal-Wallis test, also with a 5% significance level, was used for variables that did not follow normality. If significant, the Dunn test was used to test for multiple comparisons.

## RESULTS

A total of 325 HCWs aged 20-86 years were recruited. Of the 325 participants, 248 (76%) were female and the median age was 42 years (20–85; interquartile range [IQR] 31–53). Overall, 18.8% (61) of participants had positive results, with a median anti-RBD IgG quantitative titer of 64.47 BAU/mL (IQR 42.87–125.5) for the entire study group. The minimum and maximum titers obtained for the positive samples were 30.16-1,094 BAU/mL. Seropositivity was 18.1% for women and 20.7% for men. Three participants had a titer greater than 506 BAU/mL, a titer previously reported to correlate with 80% vaccine efficacy against symptomatic SARS-CoV-2 infection. The negative group included 81.2% (264) of the participants with a median anti-RBD IgG quantitative titer of 8.55 (IQR 5.5–13.92) and a maximum titer of 29.92 BAU/mL (p < 0.001).

IgG titers obtained for women were not different from those obtained for men with a seropositivity test of 62.93 (IQR 42.33-110.0 BAU/mL) and 73.02 (IQR 49.79-154.0 BAU/mL), respectively (p > 0.296).

**Figure 1:**
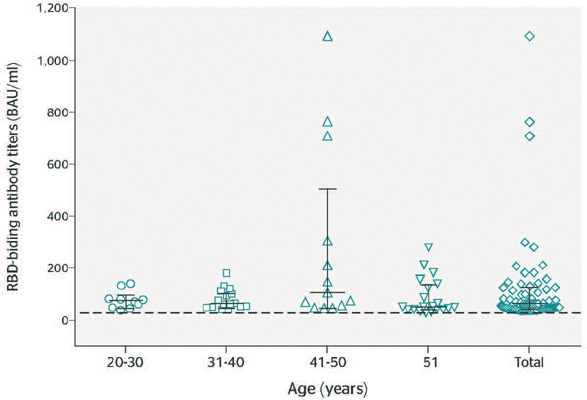
Comparison of severe acute respiratory syndrome coronavirus 2 anti-receptor binding domain (RBD) antibody titers in health care workers (HCW) by age group. Dashed line indicates the cutoff for seropositivity (30 BAU/mL). Vertical solid lines indicate the distance between the interquartile ranges. Middle horizontal solid lines indicate the median of antibodies titers, while upper and lower horizontal edges represent the 25th and 75th percentiles of antibody levels.

Table 1 and Figure 1 show the test volumes, number of positive results, and IgG titers for each age group.

**Table 1:** Detection of anti-RBD IgG seropositivity and antibody titers following 6 months of immunization with two doses of CoronaVac in HCWs

Age (years)	n	Positive n (%)	IgG titers median (BAU/mL [IQR])	OR (95%CI)	p-value*	ANOVA†
20-30	76	10 (13.15)	74.06 (46.80–94.37)	Ref.^f^	Ref.	Ref.
31-40	74	18 (24.32)	62.85 (44.90–102.00)	0.47 (0.20–1.10)	0.09	0.9900
41-50	72	13 (18.06)	105.80 (47.00–503.30)	0.68 (0.28–1.68)	0.40	0.0145
> 51	103	20 (19.42)	51.73 (40.67–137.10)	0.62 (0.27–1.43)	0.32	0.9900
Total	325	61 (18.26)	64.47 (42.87–125.50)			

BAU = biding antibodies units per mL; HCW = health care workers; IQR = interquartile range; OR = odds ratio; RBD = receptor binding domain.

*Fisher’s exact test.

^†^Multiple comparison analysis of variance (ANOVA)∕Tukey’s test and Dunn test.

## DISCUSSION

The major challenge at present is to develop predictive models of immunologic protection for COVID-19 and to define the correlates of protection in order to establish vaccination schedules.^7-9^ Data from previous studies suggest that a 28-day interval results in a more robust antibody response and longer persistence than a 14-day interval between doses.^10^ Our study found that less than 20% of the evaluated healthcare workers without prior SARS-CoV-2 infection who received two doses of vaccine were seropositive at 6 months or longer.

Recent research suggests that high neutralizing titers are required to protect against severe illness and death from circulating SAR-CoV-2 variants.^11^ We observed a decrease in antibody detection with no significant differences between different age groups. In fact, there were no statistical differences in IgG levels between age and sex. Only three participants achieved a high antibody titer, which correlates with protection according to a recently published study.^9^

In this study, two doses of an adenovirus vector vaccine were evaluated, with 28 days after the second dose and an antibody level to achieve 80% efficacy against symptoms needed to achieve an mean 506 (95% BAU/mL). Another report evaluated an immune correlate analysis of the mRNA-1273 COVID-19 vaccine trial and estimated 90% vaccine efficacy at an anti-RBD IgG level of 775 BAU/mL at that time.^12^ In our study, even the seropositive HCW would not be protected from symptomatic infection after the time period we analyzed.

A limitation of this study is the use of an immunochemiluminescence assay as a surrogate marker of the humoral immune response rather than a plaque reduction neutralization assay. Nevertheless, the assay detected all immunodominant neutralizing antibodies directed against the spike protein anti-RBD.

Other immunologic markers of cellular immunity not tested in our study may contribute to protection in previously immunized patients, even in the absence of antibody persistence. A non-peer-reviewed study of Brazilian HCW reported 50.7% efficacy of CoronaVac in preventing severe forms of SARS-CoV-2 infection in a phase III clinical trial.^13^ Another study published in China showed that HCW maintained their B-cells and T-cells specific for SARS-CoV-2 detection 5 months after two doses of Sinopharm vaccine.^14^

## CONCLUSIONS

Further research is needed to determine the durability of postvaccination antibodies in individuals, as well as other immunological markers, and to elucidate how serology tests can predict efficacy and determine the ideal timing of booster doses for population protection. Inactivated vaccines are safe, but immunogenicity may vary by age group, particularly when administered to children.
